# Dislocation of Upper End of Femur

**Published:** 1852-06

**Authors:** Frank H. Hamilton


### ART. III. — *Dislocation of Upper End of Femur, left Unreduced.*

DR. FLINT, -- It is probably no very uncommon event for a dislocated hip to be left unreduced, even where the case has been under the hands of a clever surgeon; but it is certainly not often that such cases obtain a public record. We are not over-zealous, generally, to publish our own failures, and it is hardly generous to advertise the failures of others; so that between our selfishness and our unselfishness many of the shortcomings of our art are hidden.

Fortunately Mr. Chelius, the author of a most excellent "System of Surgery," has sufficient reputation the world over to enable him to bear a portion of these failures, without injury to himself or the profession which he so eminently illustrates. I shall therefore make no apology for requesting you to record this unsuccessful attempt to reduce a dislocated hip, in which he was himself the operator.

*June* 11, 1851. John Maurer, a German, aged nineteen years, called upon me at my office, and related as follows:

"When ten years old I fell from a tree, a height of six feet, and dislocated my left hip. I was then living twelve miles from Heidelberg; and I was immediately taken there, but I did not see Mr. Chelius until the next day. He took me to the university, and, before the medical students, attempted to reduce it, but he could not. During several weeks following he tried six times, using pulleys, &c., but he never could succeed."

I find the limb shortened two inches; the knee is turned in, and the *toes out.* The dislocation is upward and backward upon the dorsum ilii. He walks rapidly and without pain or discomfort, but with a manifest halt.

Yours truly, FRANK H. HAMILTON.

